# Rehabilitation outcomes at discharge from staged community-based brain injury rehabilitation: A retrospective cohort study (ABI-RESTaRT), Western Australia, 2011–2020

**DOI:** 10.3389/fneur.2022.925225

**Published:** 2022-09-21

**Authors:** Lakkhina Troeung, Georgina Mann, Lily Cullinan, Janet Wagland, Angelita Martini

**Affiliations:** ^1^Brightwater Research Centre, Brightwater Care Group, Inglewood, WA, Australia; ^2^School of Allied Health, Faculty of Health Sciences, Curtin University, Bentley, WA, Australia; ^3^Disability Services, Brightwater Care Group, Inglewood, WA, Australia

**Keywords:** acquired brain injury (ABI), post-acute, functional rehabilitation, cognitive rehabilitation, traumatic brain injury, stroke, evaluation

## Abstract

**Objective:**

To evaluate change in functional independence, psychosocial functioning, and goal attainment at discharge from a slow-stream Staged Community-Based Brain Injury Rehabilitation (SCBIR) service in Western Australia, 2011–2020.

**Methods:**

Retrospective cohort study of *n* = 323 adults with acquired brain injury (ABI) enrolled in a post-acute SCBIR service compared against a control cohort of *n* = 312 with ABI admitted to three non-rehabilitation programs. Outcome measures were the UK Functional Independence Measure and Functional Assessment Measure (FIM+FAM), Mayo Portland Adaptability Inventory-4 (MPAI-4), and Goal Attainment Scale. Change in FIM+FAM and MPAI-4 scores and predictors of goal attainment at discharge were evaluated using multilevel mixed-effects regression.

**Results:**

Median SCBIR length of stay was 20.5 months. Rehabilitation clients demonstrated clinically significant functional gains at discharge, adjusted mean change = +20.3, *p* < 0.001 (FIM+FAM). Peak gains of +32.3 were observed after 24–30 months and clinically significant gains were observed 5 years post-admission. Individuals discharged ≤6 months had the smallest functional gains (+12.7). Small psychosocial improvements were evidenced at discharge, mean reduction = −2.9T, *p* < 0.001 (MPAI-4) but not clinically significant. 47% of rehabilitation clients achieved their goals at the expected level or higher at discharge. Compared to the control, rehabilitation clients evidenced significantly greater functional gains and psychosocial improvement but lower goal attainment. Significant predictors of goal attainment at discharge were >2 years since injury, higher cognitive function and higher emotional adjustment at admission.

**Conclusions:**

Functional recovery after ABI is a gradual and ongoing process. SCBIR is effective for functional rehabilitation post-injury but can be improved to achieve clinically meaningful psychosocial improvement.

## Introduction

Acquired brain injury (ABI) is one of the leading causes of death and disability in Australia (1). Defined as any injury to the brain after birth, ABI can be traumatic (TBI), caused by external injuries to the head, or non-traumatic (NTBI), with common causes including stroke, hypoxia, neoplasm, and encephalitis. While survival after ABI has significantly improved over the past 150 years (2), survivors still face long-term physical, cognitive, behavioral and psychosocial sequelae that can significantly impact functioning, community integration, and quality of life (3).

Early intensive inpatient rehabilitation can result in large functional gains post-injury (4, 5). However, acute gains are not always sustained post-discharge and not all patients have the readiness to engage in high intensity rehabilitation after acute recovery (5). Sustained functional recovery is more likely to be a gradual and ongoing process reflecting underlying cell repair and regeneration of damaged neural pathways over time (6). As such, shortening inpatient rehabilitation while enhancing the provision of post-acute community-based neurorehabilitation is a cost-effective strategy increasingly used in Australia, North America and the United Kingdom to promote sustained functional recovery and meaningful community participation post-injury (7, 8).

Staged community-based brain injury rehabilitation (SCBIR) is a novel model of post-acute rehabilitation for ABI developed at the *Oats Street Rehabilitation Center* in Western Australia (WA) (9), in which individuals complete slow-stream rehabilitation whilst living on-site at a community-based residential facility over 12–24 months (10). Slow-stream rehabilitation is defined as low-intensity extended-duration rehabilitation, in contrast to high-intensity, short-duration inpatient models (9). The SCBIR model involves 10 sequential stages of rehabilitation based on level of functioning, with individuals “*graduating*” through different stages with functional improvement (10). The primary focus of SCBIR is on creating a safe environment in which physical (e.g., movement, fine and gross motor tasks) and cognitive skills (e.g., planning, memory, spatial ability) are relearnt and rehearsed through real-world participation such as self-care, domestic tasks, shopping, meal preparation, and public transport training, alongside traditional multidisciplinary therapy including physiotherapy, occupational therapy, speech therapy, psychotherapy, and cognitive therapy. Psychosocial functioning is specifically targeted through community integration and participation in leisure and recreation activities such as social groups, family-based activities, engagement in community-based volunteering and employment. Additionally, the purpose-built group residences provide opportunities for day-to-day social participation and the practice of appropriate social skills.

Three early evaluations of SCBIR have been published (10–12). Two studies [*n* = 42 (10) and *n* = 92 (12)] reported significant functional improvement at discharge, with unadjusted mean gains of +15 points in Functional Independence Measure and Functional Assessment Measure (13) score. A third study reported significant psychosocial improvement in a stroke cohort (*n* = 62) (11) using the Mayo Portland Adaptability Inventory-4 (14). While these results provide preliminary support for the effectiveness of SCBIR, intervention effects may be overstated given a lack of adjustment for demographic and clinical covariates. Moreover, outcomes were not compared against any controls to evaluate the relative effectiveness of SCBIR. In all, few controlled evaluations of post-acute rehabilitation for ABI exist. An early randomized controlled trial (7) compared community-based rehabilitation for severe TBI against an information control. A second study (15) evaluated the effectiveness of an improved rehabilitation program against a historical control.

Finally, goal attainment has yet to be evaluated as a primary outcome of SCBIR. Given the clinical complexity of ABI (16), individuals present with heterogeneous rehabilitation goals and not all rehabilitation gains may be captured using individual assessments. For example, one individual may present to rehabilitation with the aim of improving motor function to return to driving, while another may aim to improve communication and social participation. Goal setting is a fundamental component of SCBIR used to design highly individualized rehabilitation programs and enable systematic evaluation of heterogeneous rehabilitation progress. Goal attainment scaling is a mathematical technique for quantifying the achievement of individualized goals and provides an overall indicator of intervention effectiveness that is not tied to any specific outcome measure (17).

As part of the ABI-RESTaRT (18) research program, this study evaluates change in functional independence, psychosocial functioning, and goal attainment, in a cohort of 323 adults with ABI who received SCBIR in WA, 2011–2020, compared against a control cohort of 312 adults with ABI admitted across three non-rehabilitation programs over the same period. Predictors of goal attainment at discharge are also investigated to inform rehabilitation planning.

## Method

### Study design and cohort definition

ABI-RESTaRT is a retrospective whole-population cohort study of all individuals aged 18–65 years who received post-acute care through Brightwater Care Group from inception in 1991 to 31 December 2020 (*n* = 1,011) (18). Inclusion criteria were English speaking and a primary diagnosis of TBI, NTBI, or eligible neurologic condition, defined by Australian Rehabilitation Outcomes Center impairment codes (19).

This study evaluates rehabilitation outcomes for a subcohort of ABI-RESTaRT members, enrolled in services between 1 January 2011 to 31 December 2020 (*n* = 635), when standardized outcome measures were introduced. The intervention cohort (“rehabilitation cohort”) is defined as all individuals enrolled in SCBIR between 1 January 2011 and 31 December 2020 (*n* = 323). The control cohort is defined as all individuals enrolled in three non-rehabilitation post-acute programs over the same period (*n* = 312; see section “Setting”).

### Setting

#### SCBIR

**Figure 1:**
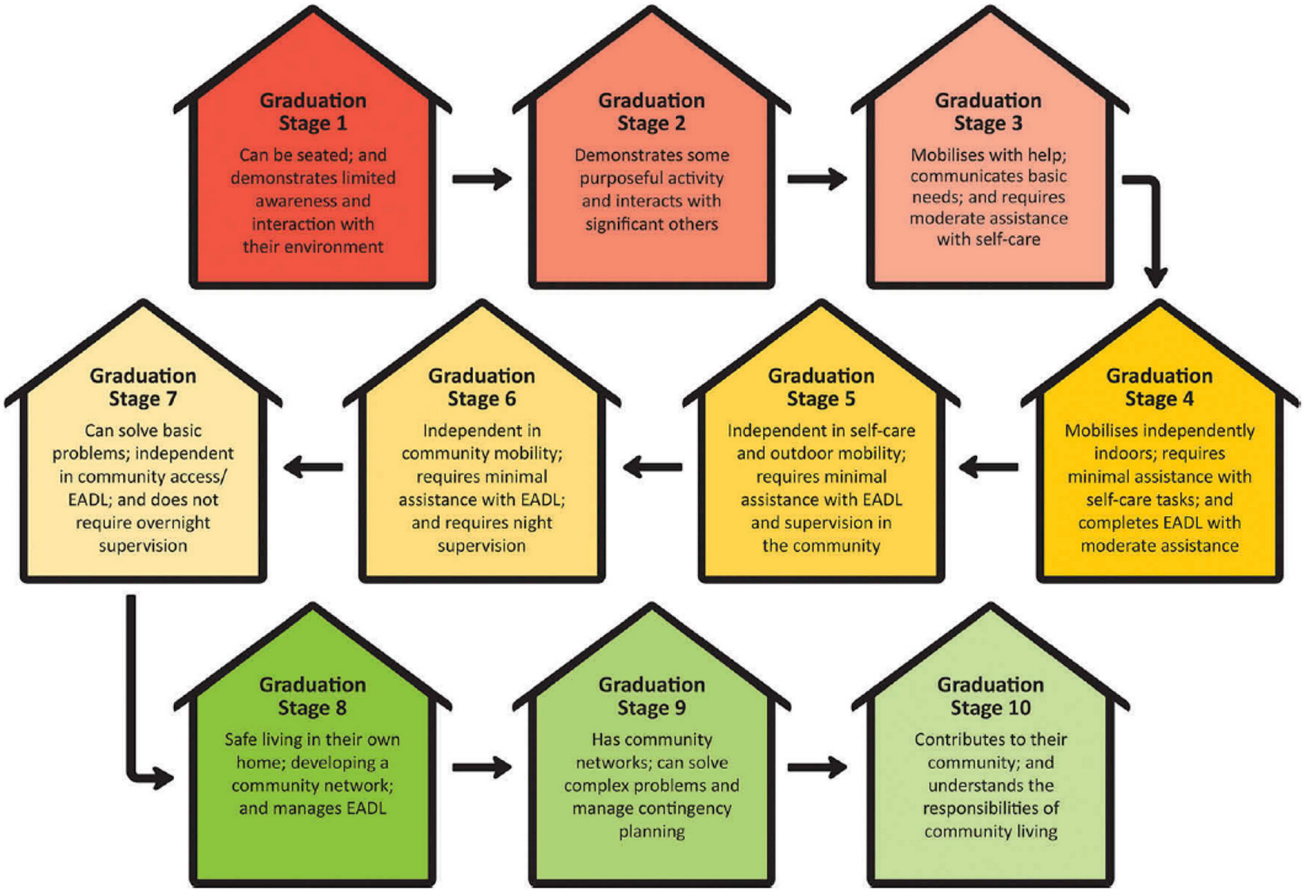
Staged community-based brain injury rehabilitation (SCBIR) graduation stages.

SCBIR is a full-time, community-based slow-stream rehabilitation program providing multidisciplinary post-acute therapy targeting functional recovery after brain injury. Clients live at the 43-bed purpose-built *Oats Street Rehabilitation Center* and receive rehabilitation within dedicated units based on their care needs. On admission, clients are allocated to a house with appropriate levels of assistance based on baseline functioning, ranging from 24-h care to full independence, and graduate through 10 stages of care with decreasing levels of support as their functioning improves (Figure 1). The program is able to support all stages of brain injury rehabilitation, from profound physical disability (including those in a minimally conscious state) to higher-level cognitive rehabilitation.

#### Control programs

##### Transitional accommodation program

Transitional accommodation program (TAP) is a transitional care service for individuals with ABI who are medically stable post-hospital discharge. Referrals must come from a WA public hospital and clients receive short-term care while being supported to seek longer-term accommodation. TAP uses a socio-medical model, involving short-term therapy, nursing and care supports to promote natural recovery but does not involve active rehabilitation.

##### Home and community care social skills

Home and community care social skills (HACCSS) is a dedicated social support program for individuals with ABI living in the community at risk of social isolation. The program operated from 2009–2019 and provided home-based social supports to individuals with ABI to engage in social activities as required.

##### Supported independent living

Supported independent living (SIL) is a supported accommodation program for individuals with ABI who require assistance to carry out activities of daily living but do not wish to engage in active neurorehabilitation. Individuals in this program live across 8 shared houses for people with disability throughout the Perth metropolitan area.

### Data sources and extraction

Outcome data were extracted from electronic medical records (EMRs) and completed on admission, review, and discharge as part of routine care. Demographic and clinical variables were extracted from EMRs and linked Hospital Morbidity Data Collection (HMDC) and Emergency Department Data Collection (EDDC) records from the WA Data Linkage System (20). Full details are presented elsewhere (18).

### Measures

#### Demographic variables

Demographic variables extracted were; age, gender, Aboriginal and Torres Islander status, relationship status, Australian Statistical Geography Standard (ASGS) Remoteness Area score (21), and Index of Relative Socio-Economic Disadvantage (IRSD) score (22). Residential postcodes prior to post-acute admission were used to generate ASGS and IRSD scores. The ASGS measures geographical remoteness based on relative access to services and divides Australian postcodes into five Remoteness Areas ranging from: 1 (*Major cities*) to 5 (*Very remote Australia*). The IRSD measures relative socioeconomic disadvantage in terms of accessibility to education, employment status and income across five quintiles ranging from 1 (*Most disadvantaged*) to 5 (*Least disadvantaged*).

#### Clinical variables

Clinical variables extracted were; ABI diagnosis, injury date, injury location (bilateral, left hemisphere, right hemisphere), acute hospitalization length of stay (“acute LOS”), time since injury to post-acute admission (“time since injury”), and prior ABI. Linked HMDC and EDDC records were used to validate ABI diagnosis and date of injury.

#### UK functional independence measure and functional assessment measure

The UK Functional Independence Measure and Functional Assessment Measure (FIM+FAM) (13) is a 30-item global measure of functional disability after brain injury assessing Motor (16 items) and Cognitive (14 items) functioning on 7-point scale ranging from 1 (*total assistance*) to 7 (*complete independence*). Relevant items are summed to produce total scores with higher scores representing greater independence.

#### Mayo portland adaptability inventory-4

The Mayo Portland Adaptability Inventory-4 (MPAI-4) (14) is a 29-item scale measuring common sequelae of ABI in the cognitive, emotional, behavioral and social domains across three subscales: Abilities (12 items), Adjustment (7 items) and Participation (8 items). Each item is scored on a 5-point scale ranging from 0 (*no limitation*) to 4 (*severe limitation*). Relevant items are summed then transformed to *T*-scores (12). Lower T-scores indicate fewer limitations (i.e., greater psychosocial functioning).

#### Goal attainment scale

The Goal Attainment Scale (GAS) (17) is a 5-item tool used to set and evaluate rehabilitation goals. At admission, the healthcare team works with the client to set 3–5 individualized goals reflecting their desired outcomes. At discharge, achievement of each goal is evaluated on a 5-point scale ranging from −2 (*a lot less than expected*) to +2 (*a lot more than expected*), with 0 representing *achievement at the expected level*. Individual scores are transformed into an aggregated GAS *T-score* to reflect overall goal attainment. *T-score*s = 50 indicate *attainment at the expected level*, scores >50 indicate *goal attainment at a level higher than expected*, and scores ≤40 indicate goals were *not achieved at the expected level*.

For this study, individual goals were categorized into 9 domains for analysis, as defined by the International Classification of Functioning, Disability and Health (ICF) Activity and Participation codes (23) (see Table S1).

### Statistical analysis

Data were analyzed using STATA 16.1. All analyses were tested against an alpha level of 0.05 (uncorrected, two-tailed). Three-level multilevel regression models with Repeated Measures (Level 1) nested within Individuals (Level 2) nested within Admission Year (Level 3) were used for all change analyses. Multilevel modeling was used to control for potential sources of bias in the data due to services changes over time and random individual variation. Individuals with completed outcome measures at admission and discharge were included in the final analysis. Individuals without a final discharge assessment (most commonly due to unplanned hospital admission) were excluded.

#### Change in FIM+FAM and MPAI-4

Change in FIM+FAM (Total, Motor, Cognitive) and MPAI-4 score (Total, Abilities, Adjustment, Participation) from admission to discharge was analyzed using a series of multilevel mixed-effects regressions fit by maximum likelihood estimation with robust standard errors. Predictor variables were: (1) time (admission vs. discharge), (2) program (SCBIR, TAP, SIL, HACCSS), and (3) the time^*^program interaction. Each analysis adjusted for 11 covariates: age at admission, gender, Aboriginal status, relationship status, ASGS score, IRSD score, diagnosis, time since injury, prior ABI, acute LOS, and post-acute LOS. Marginal effects were calculated to examine any difference in scores from admission to discharge, by program and length of stay in program. An a priori power calculation indicated that the required sample size for a multilevel model analysis to detect an anticipated medium effect size (*f* = 0.25) at a power level of 0.8 with 14 predictors was *n* = 135. Therefore, the analysis was sufficiently powered.

Clinical significance was evaluated against published Minimal Clinically Important Difference (MCID) values, which represent the smallest score change required to translate into clinically meaningful improvement (24). MCID thresholds used were 5T for MPAI-4 (25), and 8.0 (Motor) and 7.0 (Cognitive), or 15.0 for FIM+FAM total (26).

#### Goal attainment

Goal attainment at discharge was evaluated by examining the distribution of GAS *T-score*s by program and across the 9 ICF Activity and Participation domains. Predictors of goal attainment (0 = goals not achieved; 1 = goals achieved) were analyzed using a multilevel mixed-effects logistic regression. Predictors included the 11 covariates specified above plus admission outcome scores (FIM+FAM Motor, Cognitive, MPAI-4 Abilities, Adjustment and Participation).

### Ethics statement

Ethics approval was granted by the University of Western Australia Human Research Ethics Committee (HREC) (RA/4/1/9232) and WADOH HREC (RGS0000002894). Clients provided written consent for de-identified data to be used for research as part of service entry.

## Results

### Cohort characteristics

The rehabilitation cohort accounted for 51% of all admissions from 2011 to 2020 (Table 1). NTBI was most common (68%) followed by TBI (31%; see Table S2 for full diagnostic breakdown). The majority of rehabilitation clients were male (68%) with a mean age of 45.7 years at admission, which was comparable with the control (68% male, 45.2 years). The median time since injury to SCBIR admission was 8.6 months, which was significantly shorter than the control cohort (12.0 months), *p* = 0.010. The median LOS (follow-up time) in SCBIR was 20.5 months (IQR: 9.9; 34.4) which was similar to the control [22.5 months (IQR: 1.7; 63.1)], *p* = 0.5074. The rehabilitation cohort had significantly higher functional independence at admission (*M* = 128.3 ± 45.1 vs. *M* = 103.3±53.1, *p* < 0.001) but no significant difference in psychosocial functioning than the control (*M* = 48.5 ± 10.0 vs. *M* = 46.6 ± 12.0, *p* = 0.0677).

**Table 1:** Clinical and sociodemographic characteristics for the ABI-RESTaRT subcohort active between 2011–2020 (*n* = 635).

**Characteristic**	**Rehabilitation cohort, *n* = 323**	**Control cohort**		
		**TAP, *n* = 106**	**SIL, *n* = 116**	**HACCSS, *n* = 90**
**Diagnosis**, ***n*** **(%)**				
TBI	101 (31.3)	32 (30.2)	37 (31.9)	34 (37.8)
NTBI – Stroke	136 (42.1)	28 (26.4)	29 (25.0)	32 (35.6)
NTBI – Other	82 (25.4)	35 (33.0)	33 (28.5)	19 (21.1)
Neurologic	4 (1.3)	11 (10.4)	17 (14.7)	5 (5.6)
**Male**, ***n*** **(%)**	219 (67.8)	69 (65.1)	84 (72.4)	60 (66.7)
**Age at admission**, ***M*** **(SD)**	45.7 (12.5)	45.9 (13.8)	42.8 (12.0)	47.4 (14.2)
**Age at injury**, ***M*** **(SD)**	44.0 (13.3)	42.6 (15.6)	39.5 (12.3)	43.3 (15.3)
**Aboriginal**, ***n*** **(%)**	18 (5.6)	9 (8.5)	4 (3.5)	0 (0.0)
**Resides in metropolitan area**, ***n*** **(%)**	256 (84.2)	79 (82.3)	94 (83.2)	82 (91.1)
**Socioeconomic disadvantage**, ***n*** **(%)**	50 (15.5)	19 (20.0)	30 (26.8)	22 (24.4)
**Partnered**, ***n*** **(%)**	95 (29.4)	24 (22.6)	37 (31.9)	25 (27.8)
**Time since injury, median [IQR] months**	8.6 [5.2, 17.3]	6.3 [3.8, 12.0]	12.3 [7.4, 41.9]	14.9 [7.6, 30.3]
**Time since injury**, ***n*** **(%)**				
Early: <1 year	193 (61.1)	64 (66.7)	47 (43.1)	29 (33.3)
Middle: 1–2 years	61 (19.3)	13 (13.5)	16 (14.7)	22 (25.3)
Late: >2 years	62 (19.6)	19 (19.8)	46 (42.2)	36 (41.4)
**Injury location**, ***n*** **(%)**				
Bilateral	148 (45.8)	69 (65.1)	77 (66.4)	40 (44.4)
Left hemisphere	92 (28.5)	17 (16.0)	12 (10.3)	16 (17.8)
Right hemisphere	69 (21.4)	14 (13.2)	9 (7.8)	14 (15.6)
Unilateral – unspecified	4 (1.2)	0 (0.0)	13 (11.2)	6 (6.7)
Unknown	10 (3.1)	6 (5.7)	5 (4.3)	14 (15.6)
**Previous ABI**, ***n*** **(%)**	37 (11.5)	11 (10.4)	7 (6.0)	9 (10.0)
**Acute LOS, median [IQR] months**	5.1 [2.8, 7.5]	5.1 [3.2, 8.0]	6.5 [3.4, 9.2]	3.4 [1.9, 7.3]
**Post-acute LOS, median [IQR] months**	20.5 [9.9, 34.4]	19.7 [9.9, 37.9]	76.4 [19.6, 171.3]	18.1 [11.5, 39.2]
**Admission FIM+FAM**, ***n*** **(%)**	**272 (84.0)**	**86 (81.1)**	**77 (66.4)**	**20 (22.2)**
Motor, *M* (SD)	73.2 (30.7)	50.4 (34.9)	62.8 (31.5)	89.6 (16.4)
Cognitive, *M* (SD)	55.1 (18.9)	47.2 (23.8)	52.3 (22.5)	60.9 (13.7)
Total, *M* (SD)	128.3 (45.0)	97.6 (53.9)	115.1 (47.9)	150.6 (26.0)
**Admission MPAI-4**, ***n*** **(%)**	**281 (87.0)**	**84 (79.2)**	**77 (66.4)**	**81 (90.0)**
Abilities, *M* (SD)	53.4 (11.9)	57.3 (18.2)	53.1 (10.2)	46.2 (7.2)
Adjustment, *M* (SD)	52.3 (10.9)	52.7 (14.1)	49.4 (11.9)	47.6 (8.6)
Participation, *M* (SD)	42.2 (5.6)	43.5 (7.6)	43.6 (5.2)	38.2 (7.4)
Total, *M* (SD)	48.0 (9.9)	50.1 (15.2)	48.4 (7.8)	41.7 (8.0)

TBI, traumatic brain injury; NTBI, Non-traumatic brain injury; TAP, transitional accommodation program; SIL, Supported independent living; CAPB, Capacity building; HACCSS, home and community care social skills program; M, mean; SD, standard deviation; IQR, interquartile range; IRSD, Index of Relative Socioeconomic Disadvantage; Q1 or Q2, Quintile 1 or Quintile 2; LOS, length of stay. Bold values indicate statistical significance at the *p* < 0.05 level.

### Outcome measure completion at discharge

**Figure 2:**
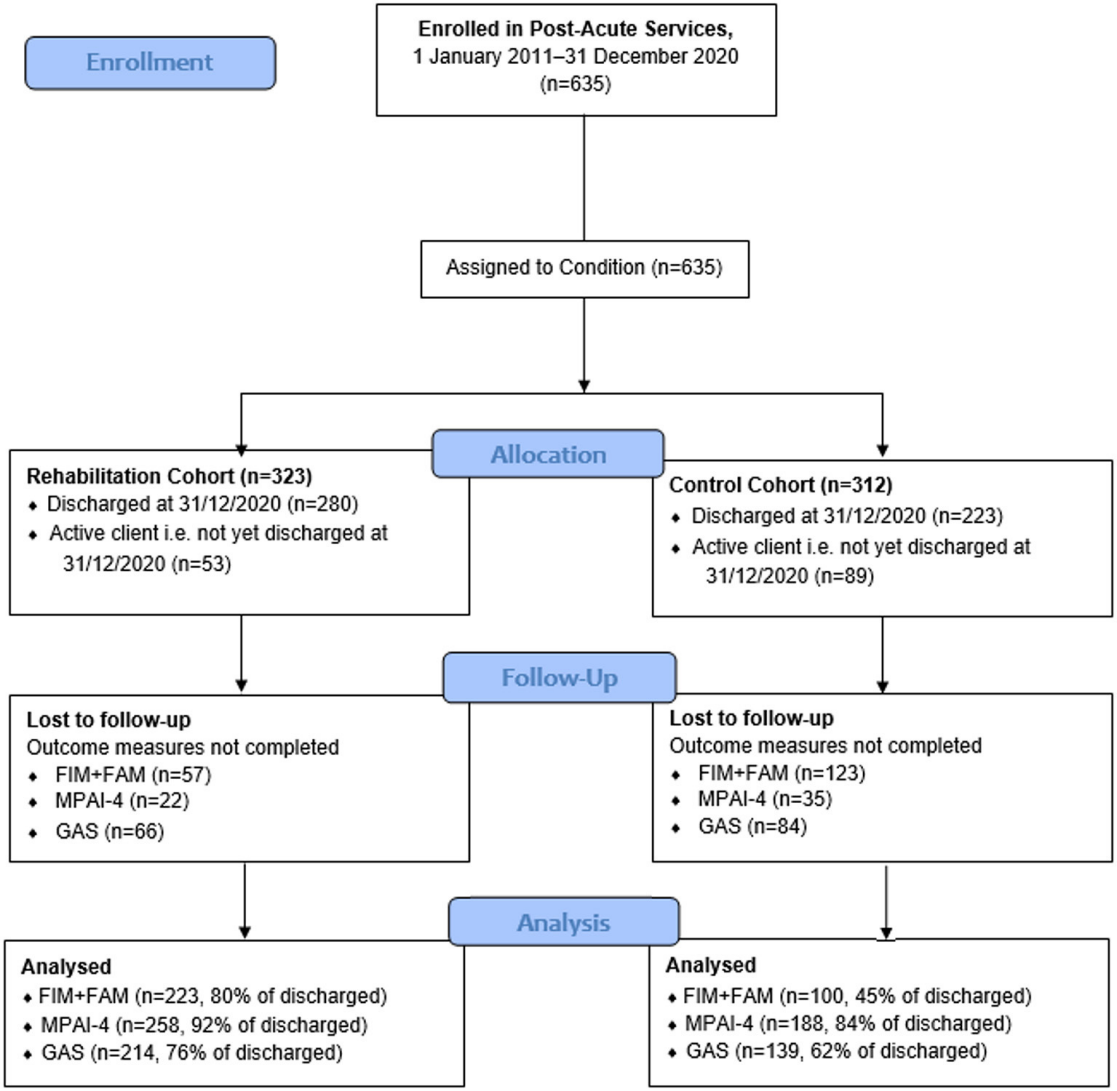
Participant flow diagram.

Figure 2 outlines participant flow and the proportion of the cohort with outcome measure data at discharge. For the rehabilitation cohort, outcome measure completion at discharge ranged from 76% (GAS) to 92% (MPAI-4). Outcome measure completion was significantly lower in the control cohort, ranging from 45% (FIM+FAM) to 84% (MPAI-4) due to differing clinical procedures across programs. There were no significant differences in demographic or clinical characteristics between participants with and without outcome measure data at discharge.

### Functional independence at discharge

On average, the rehabilitation cohort made clinically significant functional gains at discharge, with a mean adjusted gain of +20.3 (95%CI 17.1; 23.4) in FIM+FAM score, *p* < 0.001 (Table 2). Clinically significant functional gains were evident for both Motor (+11.5, 95%CI 9.9; 13.0, *p* < 0.001) and Cognitive domains (+8.8; 95%CI 7.1; 10.4, *p* < 0.001).

**Table 2:** Mean change in FIM+FAM and MPAI-4 score at discharge by program, 2011–2020.

**Program**	**Outcome measure**	***n***	**Marginal means**		**Marginal effect∧**	**95%CI**	***p*-Value**	**Clinically significant**
			**Admission**	**Discharge**				
**FIM+FAM**		323						
Rehabilitation	Motor	223	73.3	84.8	**+11.5**	**9.9, 13.0**	<0.001*	Yes
	Cognitive		58.9	67.7	**+8.8**	**7.1, 10.4**	<0.001*	Yes
	Total		128.3	148.6	**+20.3**	**17.1, 23.4**	<0.001*	Yes
TAP	Motor	49	45.8	46.7	+0.8	−0.7, 2.4	0.295	–
	Cognitive		47.5	51.9	**+4.4**	**1.8, 6.9**	<0.001*	–
	Total		90.1	95.3	**+5.2**	**1.6, 8.8**	0.004*	–
SIL	Motor	46	65.1	59.4	**−5.6**	**−8.3**, **−2.9**	<0.001*	–
	Cognitive		51.8	48.8	−3.0	−7.2, 1.2	0.157	–
	Total		116.7	108.1	**−8.6**	**−14.8**, **−2.4**	0.006*	–
HACCSS	Motor	5	96.9	105.6	+8.7	−10.7, 28.1	0.295	–
	Cognitive		61.8	64.2	+2.4	−10.4, 15.2	0.717	–
	Total		151.0	162.1	+11.1	−21.1, 43.3	0.501	–
**MPAI-4**		446						
Rehabilitation	Abilities	258	53.4	49.9	**−3.5**	**−3.6**, **−3.3**	<0.001*	–
	Adjustment		51.5	49.3	**−2.2**	**−3.3**, **−1.0**	<0.001*	–
	Participation		42.3	40.2	**−2.1**	**−2.4**, **−1.8**	<0.001*	–
	Total		48.1	45.2	**−2.9**	**−3.1**, **−2.6**	<0.001*	–
TAP	Abilities	68	54.0	54.6	+0.6	−3.3, 4.5	0.754	–
	Adjustment		50.3	51.8	+1.6	−0.3, 3.4	0.097	–
	Participation		42.5	44.2	**+1.7**	**0.9, 2.6**	<0.001*	–
	Total		47.8	49.7	**+1.9**	**0.1, 3.7**	0.034*	–
SIL	Abilities	61	54.0	54.7	**+0.7**	**0.02, 1.4**	0.044*	–
	Adjustment		49.4	52.4	**+3.0**	**1.0, 5.0**	0.003*	–
	Participation		43.8	43.7	−0.6	−1.5, 1.3	0.854	–
	Total		49.0	50.2	+2.3	−0.2, 2.7	0.100	–
HACCSS	Abilities	59	50.0	48.0	−2.0	−5.1, 1.0	0.195	–
	Adjustment		49.3	45.7	**−3.6**	**−6.3**, **−0.9**	0.009*	–
	Participation		38.6	38.1	−0.5	−3.2, 2.2	0.704	–
	Total		43.9	41.8	−2.0	−5.5, 1.4	0.248	–

∧ Marginal effect: mean difference in outcome score over time, adjusted for age at admission, gender, Aboriginal and Torres Strait Islander status, marital status, remoteness area, IRSD, diagnosis group, time since injury, prior ABI, injury location, acute LOS, post-acute LOS.

* Marginal effect statistically significant at 0.05 level.

FIM+FAM, UK Functional Independence Measure and Functional Assessment Measure; HACCSS, Home and community care social skills; MPAI-4, Mayo-Portland Adaptability Inventory; SIL, Supported independent living; TAP, Transitional accommodation program. Bold values indicate statistical significance at the *p* < 0.05 level.

#### Control cohort

The TAP cohort demonstrated small functional gains at discharge, +5.2 (95%CI 1.6; 8.8), *p* = 0.004, driven by significant cognitive improvement (+4.4, *p* < 0.001), with no change in Motor function. The HACCSS cohort also demonstrated functional improvement at discharge (+11.1, 95%CI −21.1; 43.3); however, this was non-significant due to the small number with FIM+FAM data (*n* = 5). Finally, SIL clients demonstrated significant functional decline at discharge, −8.6 (95%CI −14.8; −2.4), *p* = 0.006, which was driven by a significant decline in Motor function (−5.6, *p* < 0.001), with no change in Cognitive function.

#### Change in functional independence by LOS

**Figure 3:**
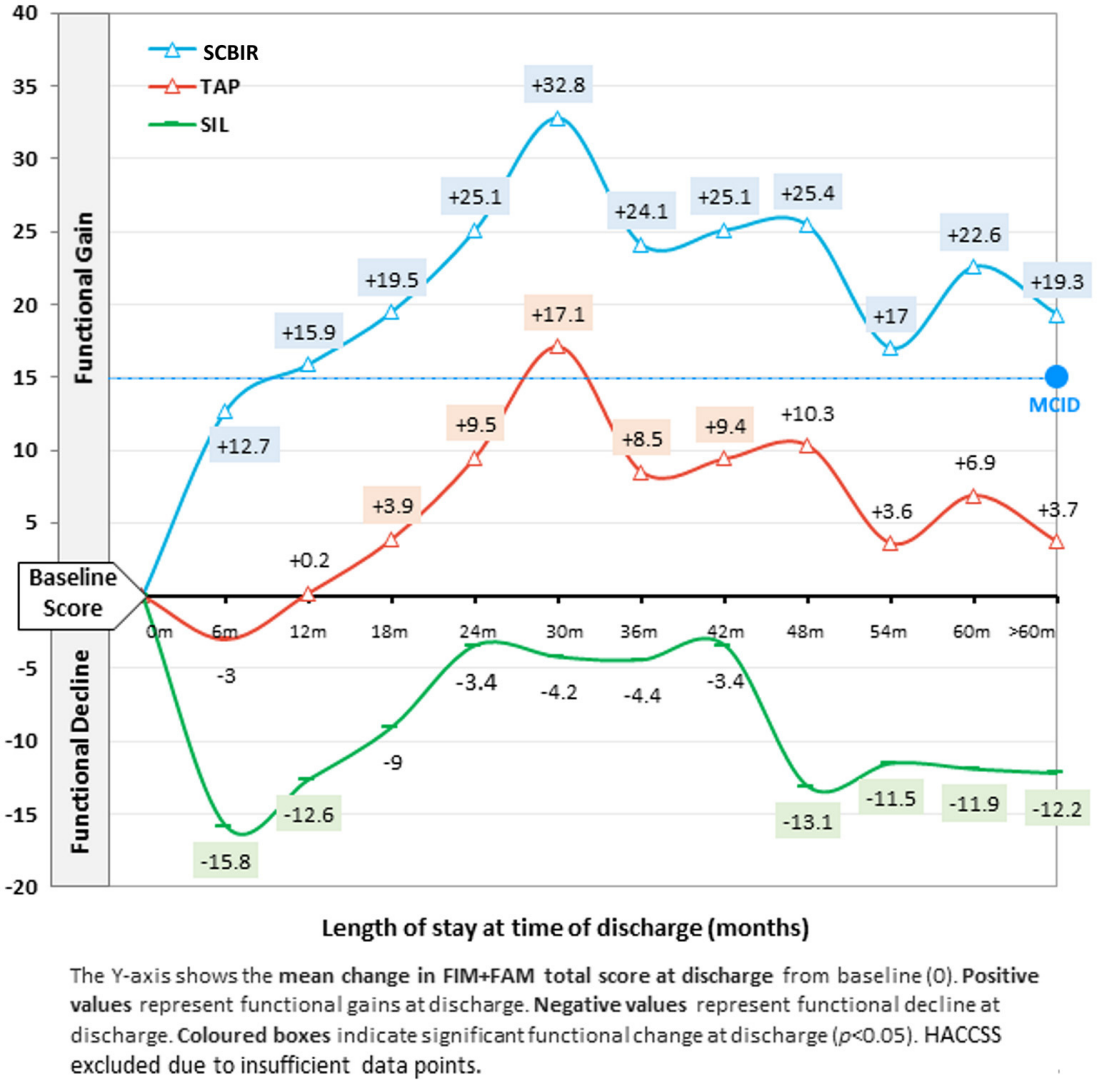
Mean change in functional independence (FIM+FAM total score) at discharge by length of stay.

Compared to the control cohort, rehabilitation clients showed significantly greater functional gains at discharge (Figure 3). On average, clinically significant functional gains were evidenced at discharge at all time points except ≤6 months. Functional gains increased with LOS, with peak gains of +32.8 observed at discharge after 24–30 months, after which a relative decline in gains was observed. However, on average, clinically significant functional gains were observed at discharge up to 5 years after admission.

Transitional accommodation program clients also showed significant functional improvement at discharge, although of lesser magnitude. Like the rehabilitation cohort, clients discharged from TAP ≤12 months did not show any functional improvements, while clients discharged between 24–30 months made clinically significant functional gains (+17.1). SIL clients showed significant functional decline at discharge. Like other programs, clients discharged ≤6 months showed the poorest outcomes (−15.8 point decline), while those who remained in SIL longer maintained their baseline level of functioning for up to 42 months, after which significant functional decline was observed.

### Psychosocial functioning at discharge

On average, rehabilitation clients made small but statistically significant improvements in psychosocial functioning at discharge, with a mean reduction of −2.9T in MPAI-4 score, *p* < 0.001. Improvement was strongest for Abilities (−3.5T), followed by Adjustment (−2.2T) and Participation (−2.1T). However, gains were not clinically significant.

#### Control cohort

The TAP cohort showed a small but statistically significant decline in psychosocial functioning at discharge, +1.9T, *p* = 0.034. The SIL cohort showed a significant decline in Adjustment (+3.0T, *p* = 0.003) and Abilities (+0.7T, *p* = 0.044) at discharge. Finally, the HACCS cohort showed significant improvement in Adjustment at discharge (−3.6T, *p* = 0.009), which represented the largest gain within any domain across all cohorts.

### Goal attainment at discharge

Just under half of the rehabilitation cohort (47%) achieved their goals at the expected level or higher at discharge (Table 3). Goal attainment was significantly lower than the control cohort (60%), *p* = 0.012, although comparable to TAP (44%). Goals related to Learning and Applying Knowledge had the highest rate of attainment (80%). General Tasks and Demands was the only domain to significantly differ between cohorts, rehabilitation cohort (74%) vs. control (91%), *p* = 0.011.

**Table 3:** Goal attainment at discharge from post-acute services, 2011–2020 (*n* = 353).

**Outcome measure**	**Rehabilitation cohort, *n* = 214**	**Control cohort**		
		**TAP, *n* = 61**	**SIL, *n* = 29**	**HACCSS, *n* = 49**
**Goals achieved**, ***n*** **(%)**				
Yes (GAS *T*-score ≥50)	100 (46.7)*	27 (44.3)	21 (72.4)	37 (75.5)
**Goal attainment category**, ***n*** **(%)**				
A lot more than expected	32 (15.0)	5 (8.2)	2 (6.9)	14 (28.6)
A little more than expected	10 (4.7)	3 (4.9)	1 (3.5)	0 (0.0)
At the expected level	58 (27.1)*	19 (31.2)	18 (62.1)	23 (46.9)
A little less than expected	90 (42.1)	28 (45.9)	7 (24.1)	12 (24.5)
A lot less than expected	24 (11.2)*	6 (9.8)	1 (3.5)	0 (0.0)
**Goal attainment by ICF domain**, ***n*** **(%)**				
Learning and applying knowledge	28 (80.0)	6 (66.7)	–	1 (100.0)
Community, social and civic life	99 (76.2)	30 (69.8)	24 (92.3)	28 (87.5)
Mobility	86 (76.1)	15 (57.7)	7 (77.8)	6 (75.0)
General tasks and demands	62 (74.7)*	16 (84.2)	6 (100.0)	30 (96.8)
Domestic life	55 (74.3)	11 (100.0)	2 (50.0)	2 (66.7)
Self-care	83 (72.8)	32 (84.2)	5 (83.3)	7 (100.0)
Interpersonal interactions and relationships	15 (71.4)	4 (66.7)	6 (85.7)	0 (0.00)
Major life areas	35 (70.0)	6 (66.7)	3 (100.0)	6 (75.0)
Communication	36 (66.7)	14 (63.6)	4 (100.0)	0 (0.0)

* Rehabilitation cohort significantly different from Control cohort at 0.05 level.

GAS, Goal Attainment Scale; HACCSS, home and community care social skills; ICF, International classification of functioning, disability and health; SIL, supported independent living; TAP, transitional accommodation program.

#### Predictors of goal attainment at discharge

Finally, Table 4 shows predictors of goal attainment at discharge. Compared with rehabilitation clients, SIL and HACCSS clients were four-times more likely to achieve goals at discharge, while there was no significant difference with TAP. Clients admitted >2 years since injury were 53% more likely to achieve goals at discharge than those admitted <1 year since injury. Clients with longer acute LOS (i.e., greater injury severity) were 3% less likely to achieve goals at discharge for every additional month of acute LOS. Interestingly, those with right hemispheric (RHS) injuries were 60% less likely to achieve goals compared to those with bilateral injuries, while there was no difference between left hemispheric and bilateral groups. Cognitive functioning at admission significantly predicted goal attainment at discharge with a 1% increase in the likelihood of goal attainment for every 1-point increase in FIM+FAM Cognitive score. Emotional adjustment at admission also significantly predicted goal attainment at discharge with a 4% increase in the likelihood of goal attainment for every 1-point increase in MPAI-4 Adjustment score. Finally, clients from remote areas were 75% less likely to achieve their goals at discharge compared with those from metropolitan areas. Those from socioeconomically disadvantaged areas were also 60% less likely to achieve their goals than those from average-to-less disadvantaged areas.

**Table 4:** Predictors of goal attainment at discharge from post-acute services, 2011–2020 (*n* = 353).

**Predictor**	**Adjusted odds ratio**	**Standard error**	**95%CI**	***p*-Value**
Age at admission	0.99	0.003	0.98, 1.00	0.399
Female	1.11	0.11	0.91, 1.34	0.301
Aboriginal	0.84	0.31	0.41, 1.74	0.639
Partnered	0.94	0.09	0.76, 1.14	0.523
**Remoteness**	**0.25**	**0.11**	**0.09, 0.57**	0.007*
**IRSD**	**0.40**	**0.09**	**0.30, 0.65**	<0.001*
**Program**				
Rehabilitation (reference)	–	–	–	–
TAP	0.64	0.12	0.40, 1.03	0.067
**SIL**	**4.01**	**1.31**	**1.67,9.05**	0.016*
**HACCSS**	**4.15**	**1.91**	**1.65, 10.2**	0.002*
**Diagnosis group**				
TBI (reference)	–	–	–	–
Stroke	0.63	0.27	0.28, 1.45	0.281
Other NTBI	0.78	0.17	0.51, 1.18	0.239
**Neurologic**	**0.13**	**0.10**	**0.03, 0.59**	0.008*
**Time since injury**				
<1 year (reference)	–	–	–	–
1–2 years	1.11	0.24	0.73, 1.69	0.611
**>2 years**	**1.53**	**0.07**	**1.40, 1.67**	<0.001*
Prior ABI	0.82	0.59	0.20, 3.34	0.782
**Injury location**				
Bilateral (reference)	–	–	–	–
Left hemisphere	0.78	0.18	0.50, 1.21	0.269
**Right hemisphere**	**0.40**	**0.08**	**0.27, 0.60**	<0.001*
**Acute LOS**	**0.97**	**0.01**	**0.95, 0.99**	0.010*
Post-acute LOS	1.02	0.03	0.97, 1.09	0.360
**FIM+FAM (baseline)**				
Motor	0.99	0.10	0.99, 1.01	0.105
**Cognitive**	**1.01**	**0.01**	**1.00, 1.02**	<0.001*
**MPAI-4 (baseline)**				
Abilities	1.02	0.01	0.89, 1.05	0.522
**Adjustment**	**0.96**	**0.01**	**0.95, 0.99**	0.001*
Participation	1.01	0.02	0.97, 1.05	0.613

* Odds ratio statistically significant at 0.05 level.

ABI, acquired brain injury; IRSD, Index of Relative Socio-Economic Disadvantage; FIM+FAM, UK Functional Independence Measure and Functional Assessment Measure; HACCSS, home and community care social skills; LOS, length of stay; MPAI-4, Mayo-Portland Adaptability Inventory; NTBI, non-traumatic brain injury; SIL, supported independent living; TAP, transitional accommodation program; TBI, traumatic brain injury. Bold values indicate statistical significance at the *p* < 0.05 level.

## Discussion

This study evaluated change in functional independence, psychosocial functioning, and goal attainment in a rehabilitation cohort of 323 adults with ABI at discharge from a post-acute SCBIR service in WA between 2011 and 2020, compared against a control cohort discharged from three non-rehabilitation programs over the same period, to determine the relative effectiveness of SCBIR in a real-world clinical setting.

Overall, our study findings add to a growing body of evidence supporting the effectiveness of SCBIR for post-acute functional rehabilitation after brain injury (10–12). The rehabilitation cohort demonstrated clinically significant gains in motor and cognitive function at discharge with a mean gain of +20.3 in FIM+FAM score. Functional gains increased with LOS and peak gains of +32.8 were observed at discharge after 24–30 months LOS, corresponding with the standard duration of SCBIR. Moreover, clinically significant gains were observed at discharge up to 5 years after admission, providing strong support for the effectiveness of slow-stream rehabilitation. Across all programs, individuals discharged earliest evidenced the poorest functional outcomes which is consistent with research showing that functional recovery post-ABI is a gradual and ongoing process resulting from regeneration of damaged neural pathways over time (6). Slow-stream rehabilitation such as SCBIR may therefore be particularly beneficial in promoting this gradual and ongoing functional recovery over time compared to short-term acute rehabilitation episodes. Outside of our service, evaluations of SCBIR are limited given it is a novel purpose-designed rehabilitation model. However, our intervention effects are similar to those reported for other post-acute community-based or outpatient neurorehabilitation services in the United Kingdom (26), and larger than reported in a previous Australian study (27).

To our knowledge, our study is the first to provide a control cohort to examine the relative change in outcomes across different post-acute programs. Overall, our results highlight the effectiveness and importance of active therapy after ABI. The rehabilitation cohort evidenced strong motor and cognitive functional gains at discharge while TAP clients showed smaller improvements in cognitive functioning at discharge. While active rehabilitation is not an overt component of TAP, it appears that significant cognitive gains can result as part of the process of planning for and seeking long-term accommodation alongside short-term therapy. In contrast, the SIL cohort showed significant functional decline at discharge, which likely represents the natural decline in functioning after ABI without intervention.

Small and statistically significant improvements in psychosocial functioning were seen at discharge from SCBIR across all three MPAI-4 domains. While improvements did not reach clinical significance, in the control cohort, TAP and SIL clients showed declines in psychosocial functioning at discharge, providing support for the relative effectiveness of SCBIR. However, overall, psychosocial gains seen in the rehabilitation cohort are smaller than reported in similar services in the literature (28), suggesting that SCBIR can be improved to better target psychosocial functioning post-ABI. In particular, SCBIR may benefit from implementing dedicated social support interventions used in HACCSS, which resulted in the largest psychosocial improvement across all cohorts, (−3.6T in Adjustment). It is also possible that the MPAI-4 used to measure psychosocial functioning in the cohort may not fully capture all relevant domains of psychosocial functioning and community integration such as family functioning. An individual's psychosocial well-being is inextricably linked to the psychosocial wellbeing of their family system, who have been shown to be profoundly affected by the occurrence of brain injury (29), including the experience of depression, anxiety, burden, social isolation, loss of income, and sacrifices in career and leisure to care for the injured members, which can cause significant family strain (30, 31). Clinically meaningful psychosocial improvements may therefore require interventions which involve the whole family system (29).

Finally, 47% of the rehabilitation cohort achieved their goals at the expected level or greater at discharge, which was significantly lower than the overall control cohort (60%), but comparable to TAP (44%). Our goal attainment rate is also higher than reported for a German post-acute inpatient rehabilitation program (31%) (32) but lower than a Norwegian home-based rehabilitation program for TBI (93%) (33). GAS scoring guidelines indicate that a goal attainment rate around 50% (or mean score of 50) is expected in large clinical populations (17), with significantly higher attainment rates suggesting that the initial goals set were too easy. Differences in goal attainment observed in our study may also in part reflect a function of time since injury, as both the rehabilitation and TAP cohorts had significantly shorter time since injury than HACCSS and SIL. Similarly, the Norwegian intervention was conducted in the chronic TBI phase. Our predictor analysis showed that individuals admitted >2 years since injury were significantly more likely to achieve goals at discharge compared with those admitted <1 year since injury, suggesting that time and opportunity are required to successfully engage with and achieve complex and major life goals after ABI (34).

Other significant predictors of goal attainment at discharge were injury location, cognitive function, emotional adjustment, remoteness and IRSD score. It is widely recognized that remoteness and socioeconomic disadvantage are associated with poorer health outcomes (35, 36). Individuals with ABI with greater socioeconomic disadvantage are more likely to have more severe injury (37) which may impact goal attainment. In addition, remoteness is associated with lower levels of health literacy (38), which may play a part in rehabilitation engagement and outcomes.

Individuals with RHS injuries were 60% less likely to achieve goals at discharge compared with those with bilateral injuries, which likely reflects the increased presence of attention, perception, learning and memory deficits after RHS injury (39). Indeed, cognitive function at admission significantly predicted goal attainment at discharge, consistent with previous literature (40). Early cognitive rehabilitation and/or training can lead to stronger rehabilitation gains at discharge (40, 41).

Finally, higher levels of emotional adjustment at admission also significantly predicted goal attainment at discharge. Symptoms of poor emotional adjustment such as anxiety, depression and anger are known to be challenging and disruptive (42) and can significantly impact rehabilitation participation and outcomes. Rehabilitation readiness is a significant predictor of outcomes (5). Early interventions to improve emotional adjustment are important to facilitate acceptance of injury and enable meaningful rehabilitation participation and better outcomes at discharge.

### Limitations

FIM+FAM completion in the control cohort was low (45%) given that functional rehabilitation is not a primary outcome of the control programs. Therefore, differences in functional outcomes between the rehabilitation and control cohort may not represent the entire control population. Additionally, outcome data was collected by different treating clinicians and may be less reliable than data collected in blinded research settings. Finally, our cohort represents individuals from a single service and therefore may not be representative of the entire Australian ABI population.

## Conclusions

Overall this study provides strong evidence for the effectiveness of SCBIR for post-acute functional rehabilitation after ABI, with clinically significant improvements in motor and cognitive functioning evidenced at discharge up to 5 years from admission. However, SCBIR can be improved to achieve clinically significant improvements in psychosocial functioning. Just under half of the rehabilitation cohort achieved their individualized goals at the expected level or higher at discharge, which is positive given the complex clinical population. Future ABI-RESTaRT research will measure post-discharge outcomes to determine whether intervention gains at discharge are sustained once the individual returns to the community.

## Data availability statement

The datasets presented in this article are not readily available due to organisational policies that restrict data sharing. Requests to access the datasets should be directed at: LT, lakkhina.troeung@brightwatergroup.com.

## Ethics statement

The studies involving human participants were reviewed and approved by University of Western Australia Human Research Ethics Committee (HREC) (RA/4/1/9232) and Western Australia Department of Health HREC (RGS0000002894). The patients/participants provided their written informed consent to participate in this study.

## Author contributions

LT designed and supervised the study, wrote the statistical analysis plan, performed data collection, analyzed the data, and drafted and revised the paper. GM performed data collection, analyzed the data, and drafted and revised the paper. LC performed the literature review and revised the draft paper. JW provided clinical and service input and revised the draft paper. AM designed and led the study and revised the draft paper. All authors have read and agreed to the statement for authors.

## Conflict of interest

Authors LT, GM, LC, JW, and AM are employed by Brightwater Care Group.

## Publisher's note

All claims expressed in this article are solely those of the authors and do not necessarily represent those of their affiliated organizations, or those of the publisher, the editors and the reviewers. Any product that may be evaluated in this article, or claim that may be made by its manufacturer, is not guaranteed or endorsed by the publisher.
