# Supplementary material for: Rehabilitation outcomes at discharge from staged community-based brain injury rehabilitation: A retrospective cohort study (ABI-RESTaRT), Western Australia, 2011–2020
Source: Front Neurol. 2022 Sep 21;13:925225. doi: 10.3389/fneur.2022.925225 (PMC9534320; doi:10.3389/fneur.2022.925225)
Supplement: Supplementary file 1 [file Table_1.DOCX]

## Appendix 1

## Table S1. ICF Activity and Participation Domains

| **Domain** | **Description / Example of goals** |
| --- | --- |
| 1. Learning and applying knowledge | - Purposeful sensory experience - Basic learning - Applying new knowledge |
| 1. General tasks and demands | - Undertaking a single task - Undertaking multiple tasks - Carrying out daily routine - Handling stress and other psychological demands |
| 1. Communication | - Receiving communication - Producing communication - Using communication devices and techniques - Participation in conversation |
| 1. Mobility | - Changing and maintaining body position - Carrying, moving, and handling objects - Walking, moving and related activities - Moving around in different locations (home, other buildings, outside) - Moving around using transportation |
| 1. Self-care | - Washing oneself - Caring for body parts - Toileting - Dressing - Eating - Drinking - Looking after one’s health |
| 1. Domestic life | - Acquisition of necessities - Household tasks - Caring for household objects and assisting others |
| 1. Interpersonal interactions and relationships | - General personal interactions - Particular personal relationship |
| 1. Major life areas | - Education - Work and employment - Economic life |
| 1. Community, social and civic life | - Community life - Recreation and leisure - Religion and spirituality - Human rights - Political life and citizenship |

ICF = International classification of functioning, disability and health
