# Supplementary material for: Rehabilitation outcomes at discharge from staged community-based brain injury rehabilitation: A retrospective cohort study (ABI-RESTaRT), Western Australia, 2011–2020
Source: Front Neurol. 2022 Sep 21;13:925225. doi: 10.3389/fneur.2022.925225 (PMC9534320; doi:10.3389/fneur.2022.925225)
Supplement: Supplementary file 2 [file Table_2.DOCX]

**Appendix 2**

**Table S2. Brain Injury Diagnoses and Australasian Rehabilitation Outcomes Centre (AROC) Impairment Codes for the ABI-RESTaRT cohort, 2011-2020**

| **AROC Code** | **Diagnosis** | **Rehab, n=323** | **TAP, n=106** | **SIL, n=116** | **HACCSS, n=90** |
| --- | --- | --- | --- | --- | --- |
| **Non-traumatic (stroke)** | | **136 (42)** | **28 (26)** | **29 (25)** | **32 (36)** |
| 1.1 | Stroke – Haemorrhagic | 36 | 6 | 7 | 8 |
| 1.2 | Stroke – Ischaemic | 95 | 21 | 21 | 22 |
| 1.0 | Stroke – Unspecified | 5 | 1 | 1 | 2 |
| **Non-traumatic (other - excluding stroke)** | | **82 (25)** | **35 (33)** | **33 (28)** | **19 (21)** |
| 2.11 | Subarachnoid haemorrhage | 26 | 7 | 8 | 3 |
| 2.12 | Anoxic brain damage | 23 | 10 | 10 | 7 |
| 2.13 | Encephalitis | 8 | 3 | 1 | - |
|  | Meningitis | 2 | 1 | 2 | 1 |
|  | Neoplasm/tumour of brain, meninges or cranial nerves | 11 | 2 | 1 | 7 |
|  | Intracranial abscess | 1 | 1 | - | - |
|  | Hydrocephalus | 1 | - | 2 | - |
|  | Toxic encephalopathy | 7 | 5 | 4 | - |
|  | Metabolic encephalopathy | 2 | 1 | 3 | - |
|  | Other non-traumatic brain dysfunction | 1 | 5 | 2 | 1 |
| **Traumatic** | | **101 (31)** | **32 (30)** | **37 (32)** | **34 (38)** |
| 2.21 | Traumatic, open injury | 22 | 6 | 6 | 6 |
| 2.22 | Traumatic, closed injury | 78 | 24 | 29 | 21 |
| 2.2 | Traumatic, unspecified | 1 | 2 | 2 | 7 |
| **Neurologic** | | **4 (1)** | **11 (10)** | **17 (15)** | **5 (6)** |
| 3.1 | Multiple sclerosis | 1 | 3 | 5 | - |
| 3.2 | Parkinsonism | - | - | 1 | - |
| 3.3 | Polyneuropathy | 1 | 1 | 1 | - |
| 3.4 | Guillain-Barré Syndrome | 1 | 1 | - | - |
| 3.8 | Neuromuscular Disorders | - | 2 | - | - |
| 3.9 | Extrapyramidal and abnormal movement disorders | - | - | - | - |
|  | Spinocerebellar disease | - | - | - | 1 |
|  | Epilepsy | 1 | - | 4 | 4 |
|  | Other neurologic and neurodegenerative disorders | - | 3 | 6 | - |
